# Polyphosphate kinases modulate *Campylobacter jejuni* outer membrane constituents and alter its capacity to invade and survive in intestinal epithelial cells *in vitro*

**DOI:** 10.1038/emi.2015.77

**Published:** 2015-12-30

**Authors:** Ruby Pina-Mimbela, Jesús Arcos Madrid, Anand Kumar, Jordi B Torrelles, Gireesh Rajashekara

**Affiliations:** 1 Food Animal Health Research Program, Ohio Agricultural Research & Development Center, Department of Veterinary Preventive Medicine, The Ohio State University, Wooster, OH 44691, USA; 2 Department of Microbial Infection and Immunity, The Ohio State University, Columbus, OH 43210, USA; 3 Center for Microbial Interface Biology, The Ohio State University, Columbus, OH 43210, USA

**Keywords:** *Campylobacter jejuni*, invasion, outer membrane constituents, poly P kinases, survival

## Abstract

*Campylobacter jejuni* is the most prevalent cause of bacterial gastroenteritis worldwide. Polyphosphate kinases 1 and 2 (PPK1 and PPK2) regulate several cellular processes, including the biosynthesis of the bacterial cell wall. Despite their importance, whether PPK1 and PPK2 modulate the composition of *C. jejuni* outer membrane constituents (OMCs) and consequently impact its interaction with host cells remains unknown. Our comparative analysis between *C. jejuni* wild type, *Δppk1,* and *Δppk2* strains showed qualitative and quantitative differences in the total OMC composition among these strains. Importantly, these OMC variations observed on the *C. jejuni* polyphosphate kinase mutants are directly related to their capacity to invade, survive, and alter the immune response of intestinal epithelial cells *in vitro*. Specifically, sub-fractionation of the *C. jejuni* OMC indicated that OMC proteins are uniquely associated with bacterial invasion, whereas *C. jejuni* OMC proteins, lipids, and lipoglycans are all associated with *C. jejuni* intracellular survival. This study provides new insights regarding the function of polyphosphate kinases and their role in *C. jejuni* infection.

## Introduction

*Campylobacter jejuni* is a foodborne pathogen responsible for causing gastroenteritis in humans worldwide. *Campylobacteriosis* cases in developing countries are underestimated because of the lack of routine surveillance programs.^1^ According to the disability-adjusted life year, approximately 7.5 million people are affected with *Campylobacter* worldwide.^2^

The *C. jejuni* cell envelope is mainly composed of a capsular polysaccharide (CPS), lipooligosaccharides, and proteins with or without *N*- or *O*-linked glycosylation.^3^ The *C. jejuni* cell envelope is thought to play an important role in immune evasion or host immune system resistance, as well as in determining the capacity of *C. jejuni* invasion of epithelial cells.^4,5,6,7^ However, to date, the exact mechanism of how *C. jejuni* modulates the host immune system to successfully colonize the host epithelia is poorly understood.

Inorganic polyphosphate (poly P) is a linear polymer of orthophosphate residues that serves as an energy source and modulates several key cellular processes in bacteria.^8,9,10^ Poly P also contributes to several important functions such as DNA entry through membrane channels, capsule composition, resistance to various stresses including nutritional and antibiotic stresses, DNA replication fidelity, growth, motility, biofilm formation, quorum sensing, bacterial signaling, stationary-phase survival, invasion and intracellular survival, and host colonization.^10,11,12,13,14^ Recent studies have shown that poly P is important for adaptation, resistance to stress, and cellular homeostasis in *C. jejuni*.^14^ However, the mechanisms through which poly P and its cognate enzymes impact *C. jejuni* pathophysiology remain largely unknown. The poly P levels in the cells are regulated mainly by poly P kinases and exopolyphosphatases (PPXs). *C. jejuni* has two poly P kinases: poly P kinase 1 (PPK1), which catalyzes the synthesis of poly P from adenosine triphosphate (ATP), and poly P kinase 2 (PPK2), which hydrolyzes poly P to generate guanosine triphosphate (GTP), a molecule known to have important roles in cell signaling and DNA, RNA, protein, and polysaccharide synthesis.^10,12,14,15,16^ In addition, PPXs degrade poly P into a smaller branch of inorganic phosphate.^17^

Previous studies have shown that *ppk1* contributes to *C. jejuni* pathogenesis and affects its tolerance to specific stresses and its stringent response.^14,15^ Furthermore, it was shown that the deletion of *ppk1* results in the decreased capacity for intracellular survival of *C. jejuni* in epithelial cells *in vitro* and a dose-dependent defect in colonization of chickens. The deletion of *ppk2* also results in impaired growth of *C. jejuni* under osmotic, nutrient, and antimicrobial stresses, as well as decreased intracellular survival in a human intestine epithelial cell line and decreased capacity for colonization of chickens *in vivo*.^16^ Additionally, the deletion of *ppk2* results in a significant decrease in poly P-dependent GTP synthesis. Reduced GTP levels can impact several cellular processes, including the composition of glycoconjugates such as glycosylated proteins.^18^ *C. jejuni* possesses both *N*- or *O*-linked glycosylation; defects in glycosylation can impact host colonization and cell invasion.^19^

Taken together, these data suggest that poly P metabolism will have significant effects on the fitness of *C. jejuni*. However, how poly P and its cognate enzymes contribute to the pathogenesis of *C. jejuni* remains unclear. Here, we investigated whether PPK1 and PPK2 participate in shaping the *C. jejuni* outer membrane constituents (OMCs) and how OMC alterations in *C. jejuni* may contribute to its infection of human epithelial cells *in vitro*. Our data indicate that PPK1 and PPK2 regulate the *C. jejuni* OMC composition, whereby variations in OMC proteins play a role in the capacity of *C. jejuni* to modulate both invasion of and intracellular survival within the host.

## Materials and methods

### Reagents

All chemical reagents used in this study were of high-grade purity from Sigma-Aldrich, St. Louis, MO, USA, unless otherwise specified. Endotoxin-free sterile water (Baxter Healthcare Corporation, Chicago, IL, USA) was used for the dialysis of all *C. jejuni* OMC fractions. Dulbecco's phosphate-buffered saline (DPBS) without CaCl_2_ and MgCl_2_ (Invitrogen, Grand Island, NY, USA) was used in all experiments. A bicinchoninic acid (BCA) Kit (Pierce, Rockford, IL, USA) was used for protein estimation, and a human CXCL8/IL-8 ELISA Kit was used for IL-8 quantification (R&D Systems, Minneapolis, MN, USA).

### Bacterial growth and OMC extraction

*C. jejuni* strains 81–176 (wild type, WT), *Δppk1*, and *Δppk2* were used in this study.^15,16^ WT, *Δppk1*, and *Δppk2* strains were grown on Mueller Hinton (MH) agar (Becton Dickinson and Company, Sparks, MD, USA) under microaerobic conditions (5% O_2_, 10% CO_2_, 85% N_2_); kanamycin was added to a final concentration of 50 µg/mL when appropriate, and the cultures incubated for 24–48 h at 42 °C. For OMC extraction, 5 g (wet weight) of *C. jejuni* WT and mutants were harvested, suspended in 50 mL of 0.1 M NaCl, and gently stirred for 48 h at 4 °C. These mixtures were centrifuged at 27 000*g*, and the supernatant containing the OMC was filtered using a 0.2 µm membrane filter (Corninig Inc., Corning, NY, USA) lyophilized, and suspended in 10 mL of endotoxin-free sterile water. The samples were then dialyzed for 72 h (three times with water changes every 12 h) using a molecular mass cut-off membrane of 500 Da (Spectrum Labs, Rancho Dominguez, CA, USA). Dialyzed samples were lyophilized, reconstituted in endotoxin free sterile water, and normalized by weight to 60 mg. The protein concentration was estimated using a BCA Kit. Samples (10 µg) were analyzed by 12% sodium dodecyl sulfate–polyacrylamide gel electrophoresis (SDS—PAGE) and stained with periodic acid-Schiff staining and silver nitrate staining to visualize glycosylations.^20^

### Fractionation of *C. jejuni* OMCs

**Figure 1:**
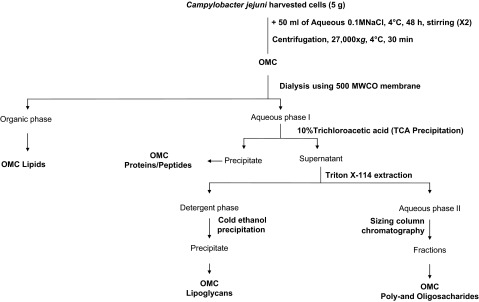
*C. jejuni* OMC extraction and fractionation. *C. jejuni* WT, *Δppk1*, and *Δppk2* OMCs were fractionated into total lipids, proteins, and poly-and oligo-saccharides, as indicated in the flowchart and as described in detail in the *Materials and Methods* section. Fractions were normalized by weight before each extraction. The obtained fractions were analyzed for their contribution to invasion by and intracellular survival of *C. jejuni* in INT-407 cells.

OMC total lipid fractions were obtained as previously described^21^ (Figure 1). Briefly, the OMC from each strain was delipidated by sequential organic solvent extractions using (i) chloroform/methanol (2:1, v/v); (ii) chloroform/methanol (1:2, v/v); and (iii) chloroform/methanol/water (10:10:3, v/v/v). Each extraction was performed by gentle shaking for 18 h at 37 °C, with centrifugation at 27 000*g* for 10 min between extractions. All organic extracts were combined to obtain the total lipid extract, and then the extracts were dried at room temperature and kept at −20 °C until use.

Delipidated OMCs from each strain was further treated with 10% trichloroacetic acid and incubated overnight at 4 °C to precipitate the total protein from the OMCs (Figure 1). The precipitate containing the OMC protein fraction was washed twice with cold acetone and dried, the total protein concentration was estimated using a BCA Kit. Samples were then aliquoted and stored at −80 °C until use.

After protein removal, the supernatant containing the lipoglycan (OMC LPG) and oligo-/poly-saccharide fractions (OMC O/P) was further extracted three times with 8% Triton X-114 (detergent) for 2 h at 4 °C, as previously described^22^ (Figure 1). This extract was further incubated in a water bath at 50 °C for 30 min to obtain a bipartition into an aqueous phase (top layer, containing OMC oligo-/poly-saccharides) and a detergent phase (bottom layer, containing OMC lipoglycans). OMC lipoglycans extracted in the detergent phase were further precipitated by adding nine volumes of 95% cold ethanol at −20 °C for 12 h. This new precipitate (OMC lipoglycans) was washed several times with cold ethanol, dialyzed, lyophilized, aliquoted, and stored at −20 °C until use. The remaining aqueous phase after detergent extraction (containing OMC oligo-/poly-saccharides) was dialyzed, lyophilized, aliquoted, and stored at −20 °C until use.

### Sugar and fatty acid analyses

Neutral sugars and fatty acid methyl esters in the total extracted OMC sample from each strain were analyzed by gas chromatography/mass spectrometry (GC/MS, Trace GC/MS ultra, Thermo Quest, Austin, TX, USA) using appropriate internal standards as previously described.^21,22,23^ Briefly, extracted OMC (normalized by 10 μg of protein content) from each strain was hydrolyzed with 2 M trifluoroacetic acid in water at 120 °C for 2 h. Using *scyllo*-inositol as the internal standard, hydrolyzed samples were reduced with sodium borodeuteride and acetylated using acetate anhydride at 120 °C for 1 h. The resulting alditol acetates were analyzed using a GC-MS (Thermo DSQII Trace coupled with GC Ultra) fitted with a Rtx-5MS column (30 m × 0.25 mm with 5 m of Integra-Guard, Restek, Bellefonte, PA, USA) with an initial temperature of 150 °C for 3 min, increased to 200 °C at 2 °C/min then to 250 °C at 40 °C/min and held for 4 min. The peak areas of the individually separated alditol acetates were used for relative quantification. Experiments were performed twice in duplicate.

### Mass spectrometry analysis of the *C. jejuni* protein fraction

Three replicates of *C. jejuni* WT, *Δppk1*, and *Δppk2* OMC total protein (10 µg protein per replicate) were trypsin digested and analyzed by capillary liquid chromatography nanospray ionization tandem mass spectrometry (capLC-NSI/MS/MS). Briefly, global protein identification was performed on a Thermo Finnigan LTQ Orbitrap mass spectrometer equipped with a microspray source (Michrom Bioresources, Inc., Auburn, CA, USA) using a µ-Precolumn Cartridge (Dionex, Sunnyvale, CA, USA) in tandem with an UltiMateTM 3000 HPLC system from LC-Packing A Dionex Co. Mobile phases A and B consisting of 50 mM acetic acid in water and 100% acetonitrile, respectively, were used with a flow rate at 2 µL/min. Mobile phase B was increased from 2% to 5% over 3 min, followed by an increase from 5% to 30% over 60 min, and then from 30% to 90% over 20 min. MS/MS data were acquired with a spray voltage of 2.2 kV and a capillary temperature of 175 °C. The full scan resolution was set at 60 000 to achieve high mass accuracy MS determination. The collision-induced dissociation fragmentation energy was set to 35%. Dynamic exclusion was enabled with a repeat count of 1 within 18 s, a mass list size limit of 500, an exclusion duration of 10 s, and a low mass width and high mass width of 30 ppm. Sequence information from the MS/MS data was processed using Mascot Daemon by Matrix Science version 2.3.2 (Boston, MA, USA) against the NCBI nr ‘*C. jejuni*'database (*vr.* 20133017, 44572 sequences). A decoy database was also searched to determine the false discovery rate (FDR), and peptides were filtered according to this FDR. The significance threshold was set at *P* < 0.05.

### Transmission electron microscopy

Transmission electron microscopy (TEM) was performed as previously described.^24,25^ Briefly, *C. jejuni* bacterial pellets were exposed to a fixative containing 3% glutaraldehyde/1% paraformaldehyde in 0.1 M potassium phosphate buffer (PB), pH 7.2. Cells were fixed for 2 h at room temperature and then embedded in 0.6% agarose; fixation was continued overnight at 4 °C with fresh fixative. After three washes with excess PB, samples were post-fixed in 1% osmium tetroxide with 1% uranyl acetate in PB for 1 h. The samples were subsequently washed three times with distilled water, dehydrated in graded ethanol, and ethanol-propylene oxide series and embedded in EM Bed812 resin following the manufacturer's instructions (Electron Microscopy Sciences, Hatfield, PA, USA). Ultrathin sections (70 nm) were prepared using a Leica EM UC6 ultra-microtome. After staining with 3% aqueous uranyl acetate for 20 min, followed by Reynolds' lead citrate for 10 min, sections were viewed using a Hitachi H-7500 transmission electron microscope at 80 kV at the Molecular and Cellular Imaging Center (http://www.oardc.ohio-state.edu/mcic), and images were recorded with an Optronics QuantiFire S99835 (SIA) digital camera.

### Cell culture assays

Cell culture studies using INT-407 cells (human embryonic intestine, ATCC CCL 6; http://www.atcc.org/products/all/CCL-6.aspx) were approved by The Institutional Biosafety Committee, The Ohio State University, under the protocol number 2007R0009AR4. For cell culture, INT-407 cells were grown in Dulbecco's minimal essential medium (DMEM) supplemented with 4 mM L-glutamine, 4.5 g/L L-glucose, and 10% fetal bovine serum (Thermo Scientific, South Logan, UT, USA) at 37 °C in a 5% CO_2_ humidified incubator. Cells on monolayers were treated with trypsin (1% trypsin, Gibco, Grand Island, NY, USA), suspended in 24 well tissue culture plates and incubated until confluent monolayers were obtained. To assess the number of INT-407 cells prior to infection, two extra wells were seeded, and the average cell number per well was determined by staining the cells with trypan blue and counting the cells with a hemocytometer under a microscope. For infection, ∼2 × 10^6^ cells per/well were used. Antibiotics were not used for culturing the INT-407 cells in this study.

### Invasion and survival assays

Different fractions from *C. jejuni* WT, *Δppk1*, and *Δppk2* were individually suspended in DMEM (150 µg/mL of the total OMC, 150 µg/mL of OMC protein fractions, 75 µg/mL of OMC lipid fractions, 75 µg/mL of OMC oligo-/poly-saccharides fractions, and 50 µg/mL of OMC lipoglycan fractions) and sterilized by filtration using 0.22 µm membrane filters (Millipore, Billerica, MA, USA). INT-407 cells (∼2 × 10^6^ cells) were pre-incubated with the *C. jejuni* total OMC and OMC fractions (1 mL/well in a 24 well tissue culture plate) at 37 °C for 1 h, followed by a challenge with mid-log phase-grown WT *C. jejuni* (∼2 × 10^8^ cells at a multiplicity of infection of 100). The bacterial numbers were determined by measuring the OD_600_ and by standard plating and determining the colony-forming units (CFU) after a serial dilution. Following infection with *C. jejuni*, plates were centrifuged at 1000*g* for 3 min at room temperature and further incubated for 3 h at 37 °C, 5% CO_2_. Infected monolayers were rinsed three times with DPBS and treated with 150 µg/mL of gentamicin for 2 h. Infected monolayers were then washed three times with DPBS and lysed with 0.1% of Triton X-100, and a 100 µL aliquot from each well was serially diluted 10-fold in DPBS and plated on MH agar in duplicate to determine the CFUs.

For the survival assay, following the invasion and after treating the infected cell monolayers with gentamicin, monolayers were rinsed twice with DPBS and incubated for an additional 24 h with 10 µg/mL of gentamicin in complete DMEM. Following incubation, infected cells were processed to assess CFUs, as described above for the invasion assay. The invasion and survival results are expressed as the mean ± SEM of experiments performed in triplicate and were repeated at least three times on different days. INT-407 cells that were not pre-exposed to the *C. jejuni* total OMC or OMC fractions but that were challenged with WT *C. jejuni* were used as a positive control for the invasion and survival assays. INT-407 cells only exposed to the *C. jejuni* total OMC but not infected with *C. jejuni* were used as a negative control.

### Assay for IL-8 secretion

Supernatants from the survival assays were collected to evaluate IL-8 release by INT-407 cells after exposure to the total OMC and OMC fractions.^26^ IL-8 production was determined using a human CXCL8/IL-8 ELISA Kit (R&D Systems) using standard curves following the manufacturer's instructions. INT-407 cells were exposed to either *C. jejuni* (MOI 100:1) for 3 h, to phorbol 12-myristate 13-acetate (PMA) for 1 h as a positive control or to medium alone as a baseline; the three treatments were compared.

### Statistical analyses

For all experiments, the data are presented as the mean ± SEM of a minimum of *n* = 2 performed at least in duplicate. Statistical analyses of data generated in this study were performed by one-way analysis of variance (ANOVA) using the Dunnett post-test in GraphPad Prism 5.0 (GraphPad Software, Inc., San Diego, CA, USA).

## Results

### Poly P kinases influence OMC composition in *C. jejuni*

**Figure 2:**
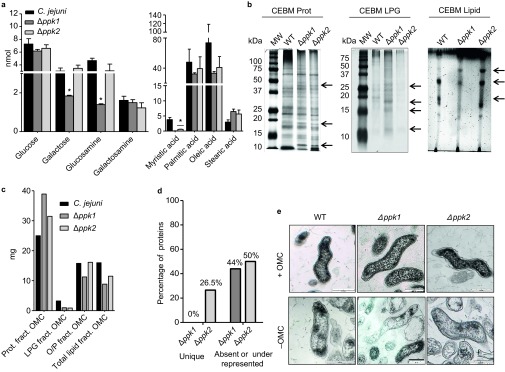
(**A**) Neutral sugar and fatty acid profile from the total OMC of *C. jejuni* WT, *Δppk1*, and *Δppk2*. Results are presented as the mean ± SEM sugar amount detected by GC and GC/MS based on 100 µg of protein. Each value is the mean of two separate experiments performed in duplicate on different days. Asterisks (*) indicate the significant difference in the sugar content compared with *C. jejuni* WT (one-way ANOVA, Dunnett's post-test, *P < 0.05). (**B**) Visualization of alterations in the OMC components produced by the deletion of poly P kinases. *C. jejuni* protein OMC fraction was analyzed in 12% SDS–PAGE and visualized by periodic acid-Schiff staining and silver nitrate staining. The *C. jejuni* lipoglycan fraction was analyzed in 15% SDS–PAGE and visualized by periodic acid-Schiff staining and silver nitrate staining. The *C. jejuni* lipid OMC fraction was analyzed by TLC using chloroform-ethanol-water-triethylamine (35:35:7:35, v/v/v/v) and visualized by charring with 10% concentrated H_2_SO_4_ in ethanol at 120°C. Arrows represent the differences in quantity and/or absence of bands between mutants compared with WT. (**C**) Quantitative analysis of the *C. jejuni* WT, *Δppk1,* and *Δppk2* total OMC material fractions. Samples were normalized by weight to 60 mg of dry OMC content. The graph represents the weight in milligrams of one biological sample extraction. (**D**) The percentage of unique and absent or underrepresented proteins present in the OMC of *C. jejuni Δppk1* and *Δppk2* compared with WT. Samples were analyzed using capLC-NSI/MS/MS. Sequence information was processed using Mascot Daemon, Matrix version 2.3.2 using *C. jejuni* database. The percentage of unique and absent or underrepresented proteins was calculated based on the overall number of proteins divided by the total number of proteins identified by capLC-NSI/MS/MS. (**E**) TEM images of *C. jejuni* WT, *Δppk1*, and *Δppk2* before (+OMC) and after treatment (−OMC) with 0.1 M NaCl. Black lines correspond to a scale bar 200 nm.

*C. jejuni* strains 81–176 (WT), *Δppk1*, and *Δppk2* were used in this study; compared to isogenic WT 81-176, these mutants have been previously shown to have no growth defect when grown in MH media.^15,16^ Neutral sugar analysis of the *C. jejuni* total OMC revealed that poly P kinases may modulate the sugar content in *C. jejuni* WT and mutant OMCs. As depicted in Figure 2a, a significant decrease (*P* < 0.05) in galactose and glucosamine was observed only in Δ*ppk1*. In contrast, the levels of glucose and galactosamine were not altered among all strains studied. The *C. jejuni* total OMC fatty acid profile also displayed variations among the strains studied; lower levels of myristic (14:0), palmitic (16:0), and oleic (18:0) acids were detected in the OMCs of the poly P mutants; however, only myristic acid levels were significantly less (*P* < 0.05) compared to the WT (Figure 2a). SDS–PAGE and thin-layer chromatography analyses of the total OMCs of poly P mutants also showed some qualitative and quantitative protein (Prot), lipoglycan (LPG), and lipid alterations compared to the WT (Figure 2b). Fractionation of the total OMC into proteins, lipids, lipoglycans, and oligo-/poly-saccharide fractions verified the observed quantitative differences (Figure 2c). Moreover, our LC/MS-MS analysis revealed qualitative differences in the total protein profiles present in the OMC protein fraction among all studied strains (Figure 2d, Table 1); compared to WT, *Δppk1,* and *Δppk2* showed 44% and 50% of proteins either absent or underrepresented, respectively, whereas 26.5% of the proteins were unique to *Δppk2*. In comparison to WT, underrepresented proteins in the *Δppk2* were γ-glutamyltransferase Ggt, SodB, PorA, and flagellins, whereas proteins such as TolB, biotin sulfoxide reductase, nitrate reductase NapA, thiol:disulfide interchange protein DsbA, FdhA, and tungstate ABC transporter were overrepresented in the *Δppk2*. In addition, the *Δppk2* completely lacked the cation ABC transporter, major antigenic peptide PEB4, PebA, methyl accepting chemotaxis protein, and KatA proteins. The *Δppk1* mutant OMC also showed several proteins such as Ggt, biotin sulfoxide reductase, PEB4, high affinity branched-chain amino acid ABC transporter, methyl-accepting chemotaxis protein, PorA, and flagellins, that were either absent or underrepresented (Table 1).

Using TEM, we further corroborated that OMC extraction does not alter the cell wall integrity of the *C. jejuni* strains studied (Figure 2e). Importantly, our viability studies assessing the ability of *C. jejuni* strains to grow on plates before and after 0.1 NaCl (0.58%) extractions showed no differences in viability when cultured in MH agar at 42 °C (Supplementary Figure S1). There are many studies showing that treatment of Gram-negative bacteria with 0.1 M NaCl removes cell envelope-bound material that maintains the integrity of the cell wall. In this regard, studies have shown that *C. jejuni* grown in a salt buffered medium at 37 °C shows no killing;^27^ only increasing the NaCl concentration in this salt buffered medium to 2%, which is 3.5-fold higher than the 0.58% used in this study, was lethal.^27^ Another study examining 44 different Gram-negative and Gram-positive bacteria established that these strains are able to grow without any deficiency after being exposed to 0.1 M NaCl.^28^ In addition, another study demonstrated that extraction of whole cells of *Escherichia coli* and *Salmonella typhimurium* with a 10-fold higher concentration (1 M NaCl) released OMC (outer material) without causing cell lysis.^29^

### OMC modifications on *ppk1* and *ppk2* mutants mediate *C. jejuni* invasion and survival in INT-407 cells

**Figure 3:**
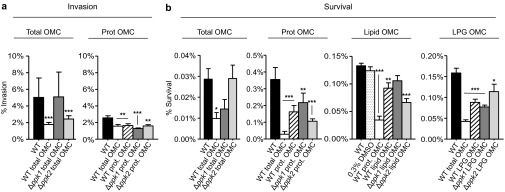
(**A**) Effect of the total OMC (150 µg/mL) and protein OMC (150 µg/mL) from WT, *Δppk1,* and *Δppk2* on *C. jejuni* invasion of INT-407 cells. (**B**) Effect of the protein (150 µg/mL), lipid (75 µg/mL), and lipoglycan (50 µg/mL) OMC fractions from WT, *Δppk1,* and *Δppk2* on *C. jejuni* survival within INT-407 cells. The total OMC was also tested with each fraction as a control using the same concentration that was used for each fraction. Results are presented as the mean ± SEM of the number of bacteria recovered after cell lysis. Each value is the mean of at least two separate experiments performed in triplicate on different days. Asterisks (*) indicate the significant difference compared to the unexposed infected INT-407 cells (one way ANOVA, ***P < 0. 001, **P < 0.01, *P < 0.05).

To assess whether differences in the total OMC composition of *ppk* mutants alter the invasion of INT-407 cells by the *C. jejuni* WT strain, INT-407 cells were exposed to total OMC extracts from WT, *Δppk1*, or *Δppk2* prior to challenge with *C. jejuni* WT. Our rationale was that components present in the WT OMC will block the invasion by WT *C. jejuni*. Alternatively, if the total OMC from *Δppk1* or *Δppk2* lacks an essential component that *C. jejuni* WT uses to invade cells, then pre-exposure of INT-407 cells to the *Δppk1* or *Δppk2* total OMC will not affect the invasion of INT-407 by the *C. jejuni* WT strain. It is possible that the incubation of the total OMC for 1 h might damage the host cells, making them permeable to gentamicin and thereby leading to less viable bacterial recovery at the end of the assay. Hence, we performed trypan blue staining at the end of the 1 h incubation to test the viability of the host cells after exposure to OMC. The results indicated that neither WT nor mutant OMC had an effect on the host cell viability (Supplementary Figure S2). As expected, our results revealed that the total OMC from WT were capable of significantly decreasing the invasion by *C. jejuni* WT by ∼300% (Figure 3a, white bar, ***P < 0.001) compared to the infected cells that were not pre-exposed to the WT total OMC (Figure 3a, black bar). However, when INT-407 cells were exposed to either the *Δppk1* total OMC (Figure 3a, dark gray bar) or the *Δppk2* total OMC (Figure 3a, light gray bar), no significant effects were observed on the invasion capacity of *C. jejuni* WT. These results imply that *Δppk1* and *Δppk2* lack component(s) on their OMC that participate in the invasion of the bacterium into human intestinal epithelial cells.

Next, we assessed which OMC fraction (lipid, protein, lipoglycans, and/or oligo-/poly-saccharides) was responsible for the decreased capacity of invasion by *C. jejuni* observed in the INT-407 cells pre-exposed to the total OMC derived from the WT strain (Figure 3a, Prot OMC). Only when INT-407 cells were exposed to the OMC protein fraction from any of the strains studied was a significant decrease 120% in bacterial invasion observed compared with the unexposed infected cells (Figure 3a, Prot OMC, **P < 0.01, ***P < 0.001). In contrast to the total OMC fraction, pre-exposure to the Prot OMC fraction of *Δppk1* and *Δppk2* resulted in decreased invasion of INT-407 cells by WT *C. jejuni*. This phenomenon could be due to interference or masking of the proteins involved in invasion by other components of *Δppk1* or *Δppk2* OMC such as lipoglycans or proteins present in the total OMC fraction and/or also to a difference in the relative abundance of functional protein component(s) present in the Prot OMC fraction compared to the total OMC of the *Δppk1* and *Δppk2* mutants. These data suggest that the OMC protein fraction from WT, *Δppk1*, and *Δppk2* mediate *C. jejuni* invasion into INT-407 cells. No significant differences were found in bacterial invasion when exposed to the lipid, lipoglycan, or oligo-/poly-saccharides fractions of any of the strains studied (Supplementary Figure S3a).

The role of poly P kinases in *C. jejuni* intracellular survival was also determined after 24 h of incubation with gentamicin to kill extracellularly attached bacteria. Here, we hypothesized that the lack of certain OMC components of mutants (but present in WT) that are key mediators of host cell receptor recognition and downstream signaling events might compromise the intracellular survival ability of WT *C. jejuni* in host cells when exposed to WT OMC but not when exposed to mutants' OMC. Pre-exposure of INT-407 cells to the *C. jejuni* WT total OMC fraction resulted in a 230% reduction in intracellular survival (*P <0.05) compared to the INT-407 cells that were not pre-exposed to the *C. jejuni* WT total OMC (Figure 3b). Similarly, pre-exposure to the OMC protein fractions from WT, *Δppk1*, and *Δppk2* resulted in a significant decrease in bacterial intracellular survival (Figure 3b). Conversely, only pre-exposure to the OMC lipid fraction from *Δppk2* or the OMC lipoglycan fraction from WT, *Δppk1*, and *Δppk2* showed a significant reduction in the % of intracellular bacteria counts (***P < 0.001, **P < 0.01, *P < 0.05; Figure 3b). No significant differences were found in bacterial survival when exposed to the oligo-/poly-saccharides fraction of any of the strains studied (Supplementary Figure S3b).

### OMC modifications on *ppk1* and *ppk2* mutants modulate IL-8 production in INT-407 cells

**Figure 4:**
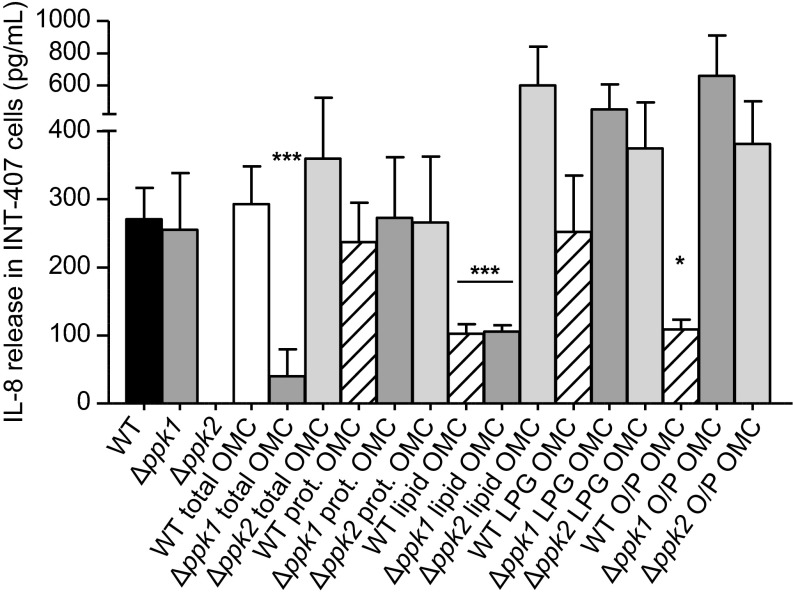
IL-8 release by INT-407 cells during *C. jejuni* intracellular survival after exposure to the OMC and the OMC fractions. INT-407 cells were incubated with *C. jejuni* OMC fractions for 1 h, infected with *C. jejuni* WT, and incubated for an additional 24 h. IL-8 release was assessed using ELISA. The results are presented as the mean ± SEM of the quantity of IL-8 released by INT-407 cells detected by ELISA. Each value is the mean of two separate experiments performed in triplicate (one way ANOVA, ***P <0.001, *P <0.05).

We next evaluated the IL-8 release by intestinal epithelial cells in the presence of the total OMC and its fractions (proteins, lipids, lipoglycans, and oligo-/poly-saccharides) from WT, *Δppk1*, and *Δppk2*. It has been previously reported that exposure of INT-407 cells with *C. jejuni* for 24 h induces IL-8 production;^26^ hence, in this study, INT-407 cells infected with live *C. jejuni* were used as a positive control. IL-8 production was observed at different levels between the total OMC and each of the OMC fractions studied (Figure 4). Conversely, IL-8 production was similar between the strains for any given OMC fraction, with the exception of the *Δppk1* total OMC and lipid OMC fractions, as well as the WT OMC oligo-/poly-saccharide and lipid OMC fractions, where lower levels of IL-8 production were observed (***P < 0.001, *P < 0.05; Figure 4). Surprisingly, IL-8 secretion was not induced by the *Δppk2* strain (Figure 4).

Overall, these results support the concept that poly P kinases play an important role in the modulation of *C. jejuni* virulence by altering its OMC composition, leading to the modulation of IL-8 secretion by INT-407 cells.

## Discussion

*C. jejuni* possesses a highly variable OMC composition, including molecules such as lipids, oligo- and poly-saccharides, and lipoproteins besides mechanisms of *N*-glycosylation in the periplasm that affects *C. jejuni* periplasmic and surface proteins and *O*-glycosylation mechanisms of the flagellin, which are critical for structural integrity and the pathogen–host interaction.^30,31,32,33^ Poly P kinases have been shown to be associated with *C. jejuni* virulence.^14,15,16^ However, there is limited information about how poly P kinases interfere with the mechanisms of regulation associated with *C. jejuni* outer structures, including the OMC. Here, we provide insights about the contribution of poly P kinases (PPK1 and PPK2) in shaping the composition of the *C. jejuni* OMC and how these modifications play a role in invasion and survival, as well as in modulating the secretion of IL-8. Here, we purified and confirmed the existence of variability in sugar and fatty acid composition. Overall, the variability in the amounts of OMC proteins, lipids, and carbohydrates among WT, *Δppk1*, and *Δppk2* strains indicated the important role of poly P kinases in the plasticity of the *C. jejuni* OMC composition.

Many virulence factors have been suggested to be associated with the invasion and survival in *C. jejuni* in different cell lines *in vitro*.^34^ Some of these virulence factors, such as LOS, CPS, flagellins, chemotactic proteins, *O-* and *N-*linked protein glycosylation systems and lipoproteins, have been linked to the mechanism regarding the invasion of epithelial cells *in vitro*.^30,35,36,37^ Thus, it is not surprising that there are complex interactions occurring between *C. jejuni* and host cells, involving several molecular structures from its cell envelope. Moreover, studies on the function of poly P kinases revealed the importance of these enzymes as regulators for metabolic processes, including biosynthesis of the bacterial cell envelope.^38^ Specifically, compared to WT, the *C. jejuni ppk2* mutant is defective in poly P-dependent GTP generation and also displays a significant increase in poly P-dependent ATP generation, suggesting that PPK2 plays a role in maintaining the intracellular nucleotide pool.^16^ Because polysaccharides are synthesized via guanosine 5′-diphosphate -linked sugars as intermediates, the need for nucleoside triphosphates (NTPs), particularly GTP, to generate glycoconjugates is substantial.^18^ Because PPK2 affects GTP and the NTP pools, PPK2 might also impact glycosylation profiles in *C. jejuni*. For example, several proteins that were absent in *ppk2* mutant OMC (methyl-accepting chemotaxis protein -type signal transduction protein, major antigenic peptide PEB-cell binding factor, etc.) were found to carry potential N-linked glycosylated sites, as predicted using GLYCOPP V1.0 (http://www.imtech.res.in/raghava/glycopp/submit.html). These findings support our study and suggest that PPK1 and PPK2 are involved in varying the OMC composition of *C. jejuni*, thus defining their role in virulence.

Our results identified the existence of *C. jejuni* OMC components that play a major role in invasion and survival, which we speculate are directly involved in host cell receptor recognition and in initiating the signaling events that lead to invasion of and survival within the intestinal epithelium. In this regard, both *Δppk1* and *Δppk2* OMCs provide us with a unique tool to identify what specific components within the *C. jejuni* WT OMC fraction mediate invasion and, importantly, which host cell receptors and signaling mechanisms are involved. In this regard, to assess the association of poly P kinases of *C. jejuni* with virulence, we saturated cell receptors related to invasion and/or intracellular survival processes by persistent incubation of INT-407 cells with the *C. jejuni* total OMC and OMC fractions prior to infection. Our rationale was that components present in the WT OMC would block invasion or alter intracellular signaling events by WT *C. jejuni*; however, if OMC from the *ppk* mutants lacks a component that *C. jejuni* uses to invade and survive in cells, then pre-exposure to the total OMC from the *ppk* mutants will affect the invasion and survival. Our results indicated that poly P kinases have an impact on the total OMC, altering invasion and intracellular survival in *C. jejuni*. We further defined the OMC protein and lipoglycan fractions as capable of interacting with the receptors used by *C. jejuni* to invade human intestinal epithelial cells, thus implying their role in receptor recognition. In this context, previous studies have described the association of several proteins (PEB1 (a major *C. jejuni* cell adhesion molecule), including lipoproteins such as JlpA (a *C. jejuni* adhesion promoting bacterial interaction with host cells) and CPS, with adhesion and invasion processes in *C. jejuni*.^39,40^ Our proteomic results concur with above studies and that found the presence of PEB-cell binding factors (PebA and PEB4) in the *C. jejuni* WT and *Δppk1* total OMC but not in the *Δppk2* total OMC^39^ (Table 1). Similarly, JlpA was present in *C. jejuni* WT and *Δppk2* but not in the *Δppk1* total OMC. In addition, several proteins were absent in *Δppk1* or *Δppk2* OMC compared to WT OMC (i.e. flagellins, Ggt, biotin sulfoxide reductase, catalase), whereas some proteins were over- or underrepresented in these mutants (i.e. MOMP, DsbA periplasmic iron and amino acid binding proteins). The lack of CJJ81176_1128 (encoding a methyl chemotaxis protein, *tlp8*) results in decreased invasion and intracellular survival.^41^ In our study, the CJJ81176_1128 was either absent or underrepresented in both *Δppk* mutants. Similarly, SodB was underrepresented in the *Δppk2* OMC; the *sodB* mutant of 81-176 has been shown to have a significant defect in the invasion of INT407 cells.^42^ These data further suggest a participating role for the poly P kinases in determining the *C. jejuni* OMC protein distribution and/or content and thereby modulating invasion by and intracellular survival of *C. jejuni*.

The role of OMC lipids in bacterial virulence has been studied in *Mycobacterium tuberculosis* and *Francisella novicida,* among other pathogens.^43,44,45^ Likewise, *C. jejuni* OMC contains lipid-containing components such as lipoglycans, which play important roles in binding, adhesion, and invasion of human epithelial cells *in vitro*.^35,44^ Our results using OMC lipid and lipoglycan fractions from *C. jejuni* WT and poly P kinases' mutants indicate that missing OMC lipoglycans and lipids from *ppk1* and *ppk2* OMC, respectively, are involved in *C. jejuni* survival. This outcome suggests the importance of poly P kinases as virulence factors associated with *C. jejuni* intracellular survival. Incubation of INT-407 cells with live *C. jejuni* also leads to the release of IL-8, and its production is directly proportional to the invasive ability of *C. jejuni* strains.^26^ Our data indicate that INT-407 cells incubated with the *C. jejuni* WT and Δ*ppk1* strains lead to IL-8 secretion, whereas there was no detectable production of IL-8 when cells were exposed to the Δ*ppk2* strain. In this context, *C. jejuni* secretes virulence factors, such as outer membrane vesicles and *Campylobacter* invasion antigens proteins associated with host cell signaling events that promote epithelial cell invasion, inflammatory response stimulation, and intracellular survival.^46,47^ According to our results, this discrepancy in the lack of IL-8 stimulation by the *Δppk2* strain in INT-407 cells could be explained by (i) deletion of *Δppk2* altered the OMC protein composition and disposition (Table 1), such that host cell receptors are not able to effectively recognize OMC proteins and thus fail to induce the secretion of IL-8, and/or (ii) that an essential protein for *C. jejuni* host cell recognition and subsequent stimulation of the immune response is missing or in low abundance in the Δ*ppk2* OMC.

Conversely, studies using sub-fractions of the *C. jejuni* cell envelope extracted by sonication and cell envelope sub-fractions inactivated with formalin have demonstrated the inability of these sub-fractions to stimulate IL-8 in INT-407 cells, suggesting that INT-407 cells require live *C. jejuni* 81-176 WT to release IL-8.^26^ However, our results suggest that the *C. jejuni* total OMC and some of the OMC fractions studied are capable of inducing the secretion of IL-8. This finding may be because the OMC was obtained solely by the disruption of non-covalent interactions using 0.1 M NaCl. Another plausible explanation is that in our studies, host cells are exposed to individual OMC fractions; thus, we are enriching for a specific pool of *C. jejuni* OMC components. This notion may explain some of the differences observed in invasion, survival, and IL-8 production between the total OMC and a particular OMC fraction.

In summary, this study adds to the known function of poly P kinases in *C. jejuni* virulence, providing the basis for further investigations in determining which specific OMC components are responsible for invasion, intracellular survival, and immune response generation. In addition, future studies are required to identify the host cell receptor(s) involved in these processes and to elucidate the possible trafficking and signaling pathways that these receptors can activate. Furthermore, *in vivo* assays will be required to evaluate the implications of OMC and OMC fractions in *C. jejuni* pathogenesis to further establish the basis of drug development targeting *C. jejuni* PPK1 and PPK2.

## Figures and Tables

**Table 1:** Identification of proteins presented in the *C. jejuni* protein OMC fractions by capLC-NSI/MS/MS.

Locus tag/gene	Protein name	Molecular mass (kDa)	WT	Δ*ppk1*	Δ*ppk2*
CJJ81176_0067; *ggt*	Gamma-glutamyltransferase	60	10	0	2
CJJ81176_0075	Cytochrome c family protein	39	6	0	3
CJJ81176_0080; *flgD*	Flagellar basal body rod modification protein	31	0	1	5
CJJ81176_0097; *fliY*	Flagellar motor switch protein FliY	30	0	0	3
CJJ81176_0147; *tolB*	Translocation protein TolB	45	3	0	6
CJJ81176_0179	Cation ABC transporter, periplasmic cation-binding protein	35	2	10	0
CJJ81176_0205; *sodB*	Superoxide dismutase, Fe	25	17	26	7
CJJ81176_0211	Iron ABC transporter, periplasmic iron-binding protein	37	93	135	93
CJJ81176_0291	Biotin sulfoxide reductase	93	1	0	8
CJJ81176_0325; *modA*	Molybdenum ABC transporter, periplasmic molybdenum-binding protein	24	3	2	3
CJJ81176_0354; *ndk*	Nucleoside diphosphate kinase	15	1	5	0
CJJ81176_0382; *ccpA-2*	Cytochrome C551 peroxidase	37	0	0	3
CJJ81176_0624	Major antigenic peptide PEB4	30	68	34	0
CJJ81176_0642	Phosphate ABC transporter, periplasmic phosphate-binding protein, putative	36	0	0	6
CJJ81176_0801; *napA*	Nitrate reductase catalytic subunit	105	4	12	65
CJJ81176_0836	Amino acid-binding protein	29	16	2	0
CJJ81176_0883	Thiol:disulfide interchange protein DsbA, putative	26	0	0	12
CJJ81176_0928; *pebA*	Bifunctional adhesin/ABC transporter aspartate/glutamate-binding protein	28	135	122	0
CJJ81176_0974	Putative lipoprotein	16	4	1	0
CJJ81176_1002; *jlpA*	Surface-exposed lipoprotein	42	5	0	4
CJJ81176_1016	Hypothetical protein	21	0	0	24
CJJ81176_1037	High affinity branched-chain amino acid ABC transporter, periplasmic amino acid-binding protein	40	0	0	22
CJJ81176_1038	High affinity branched-chain amino acid ABC transporter, periplasmic amino acid-binding protein	81	46	20	0
CJJ81176_1128; *tlp8*	Methyl-accepting chemotaxis protein	48	20	11	0
CJJ81176_1242; *htrA*	Protease DO	51	2	2	4
CJJ81176_1275; *porA*	Major outer membrane protein	46	83	15	67
CJJ81176_1308; *accB*	Acetyl-CoA carboxylase, biotin carboxyl carrier protein	16	0	2	4
CJJ81176_1338	Flagellin	60	156	0	105
CJJ81176_1339	Flagellin	60	215	40	157
CJJ81176_1387; *katA*	Catalase	50	9	11	0
CJJ81176_1503; *fdhA*	Formate dehydrogenase, alpha subunit, selenocysteine-containing	104	0	0	6
CJJ81176_1525	Tungstate ABC transporter, periplasmic tungstate-binding protein, putative	30	0	0	39
CJJ81176_1604	Hemin ABC transporter, periplasmic hemin-binding protein, putative	29	8	8	0
CJJ81176_1650	Hypothetical protein	19	0	0	20

Numbers in each strain represent the relative protein abundance (based on spectral counts) from three replicates of 10 µg of outer material analyzed. Only proteins having spectral counts of more than two in at least one of the strains analyzed are included.
