# Supplementary material for: Polyphosphate kinases modulate Campylobacter jejuni outer membrane constituents and alter its capacity to invade and survive in intestinal epithelial cells in vitro
Source: Emerg Microbes Infect. 2015 Dec 30;4(12):e77–. doi: 10.1038/emi.2015.77 (PMC4715166; doi:10.1038/emi.2015.77)
Supplement: Supplementary Figure S1 [file emi201577x1.pdf]

Supplemental Figure 1

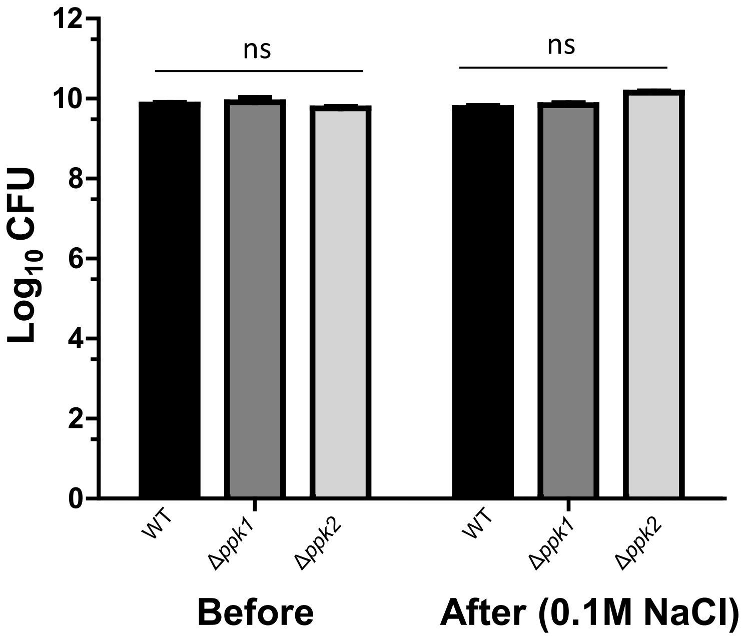

Supplemental Figure S1 Effect of 0.1 M NaCl on the viability of WT, *Δppk1* and *Δ?ppk2 C. jejuni* strains. Overnight grown cultures were harvested in MH broth and washed once by centrifuging at 10 000 *g* for 5 min. The cultures were suspended in MH broth to adjust the OD_{600nm} to 1.0 in a 14 mL culture tube. Before treatment, 100 µL of the adjusted OD cultures was used to determine the CFU by a 10-fold serial dilution. Culture tubes were incubated at 4° C in microaerophilic conditions for 48 h. After incubation, CFUs were determined by plate counting. Results are presented as the mean±SEM of two independent experiments with duplicate samples in each experiment. \`ns' indicates non-significant (One way ANOVA).
