# Supplementary material for: Polyphosphate kinases modulate Campylobacter jejuni outer membrane constituents and alter its capacity to invade and survive in intestinal epithelial cells in vitro
Source: Emerg Microbes Infect. 2015 Dec 30;4(12):e77–. doi: 10.1038/emi.2015.77 (PMC4715166; doi:10.1038/emi.2015.77)
Supplement: Supplementary Figure S2 [file emi201577x2.pdf]

Supplemental Figure 2

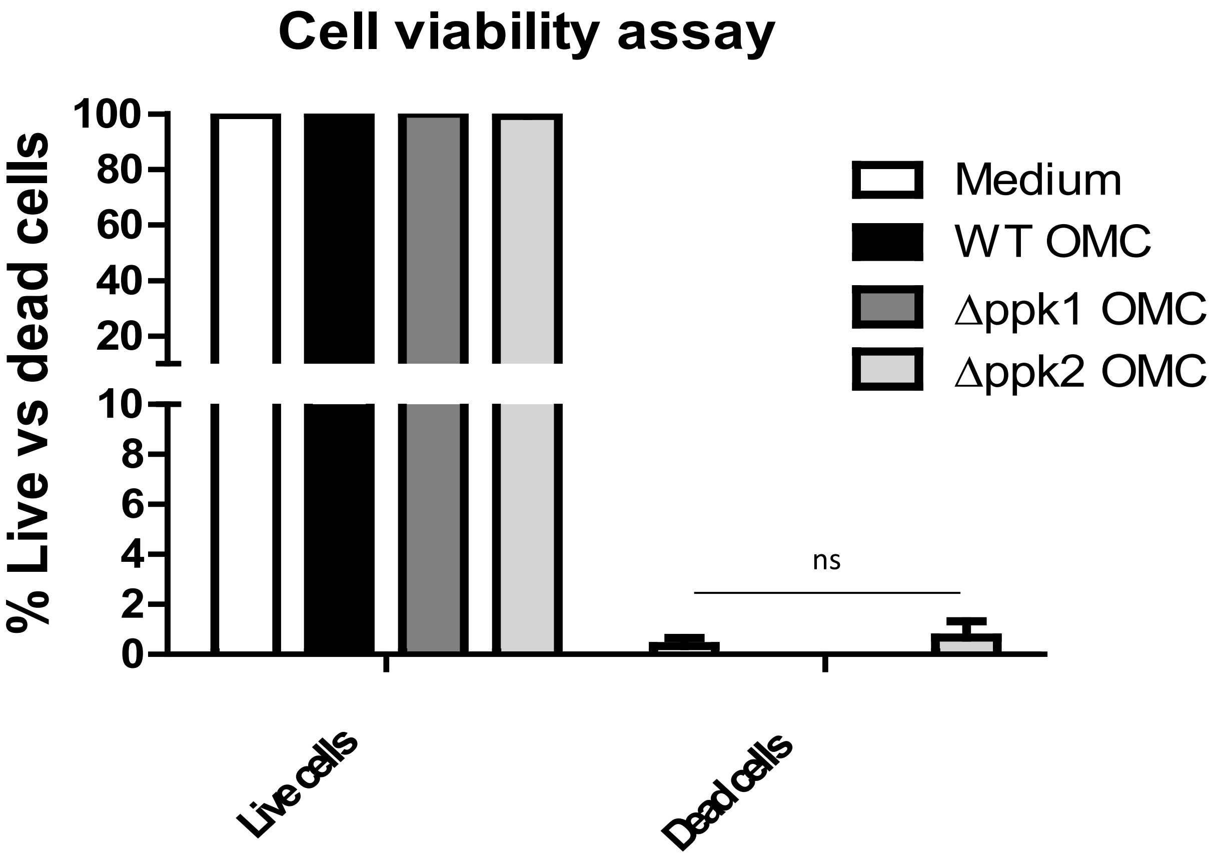

Supplemental Figure S2 Determining the effect of the total OMC from WT, *Δppk1* and *Δppk2* on INT-407 cell viability. INT-407 cell monolayers were incubated with the OMC from *C. jejuni* wild type and mutants (150 µg/mL) for 1 h in triplicate wells, and the viability of the cells was determined using trypan blue staining. Results are presented as the mean±SEM of the number of live and dead cells, where `ns' indicates not significant (one way ANOVA).
