# Supplementary material for: Polyphosphate kinases modulate Campylobacter jejuni outer membrane constituents and alter its capacity to invade and survive in intestinal epithelial cells in vitro
Source: Emerg Microbes Infect. 2015 Dec 30;4(12):e77–. doi: 10.1038/emi.2015.77 (PMC4715166; doi:10.1038/emi.2015.77)
Supplement: Supplementary Figure S3 [file emi201577x3.pdf]

Supplemental Figure 3

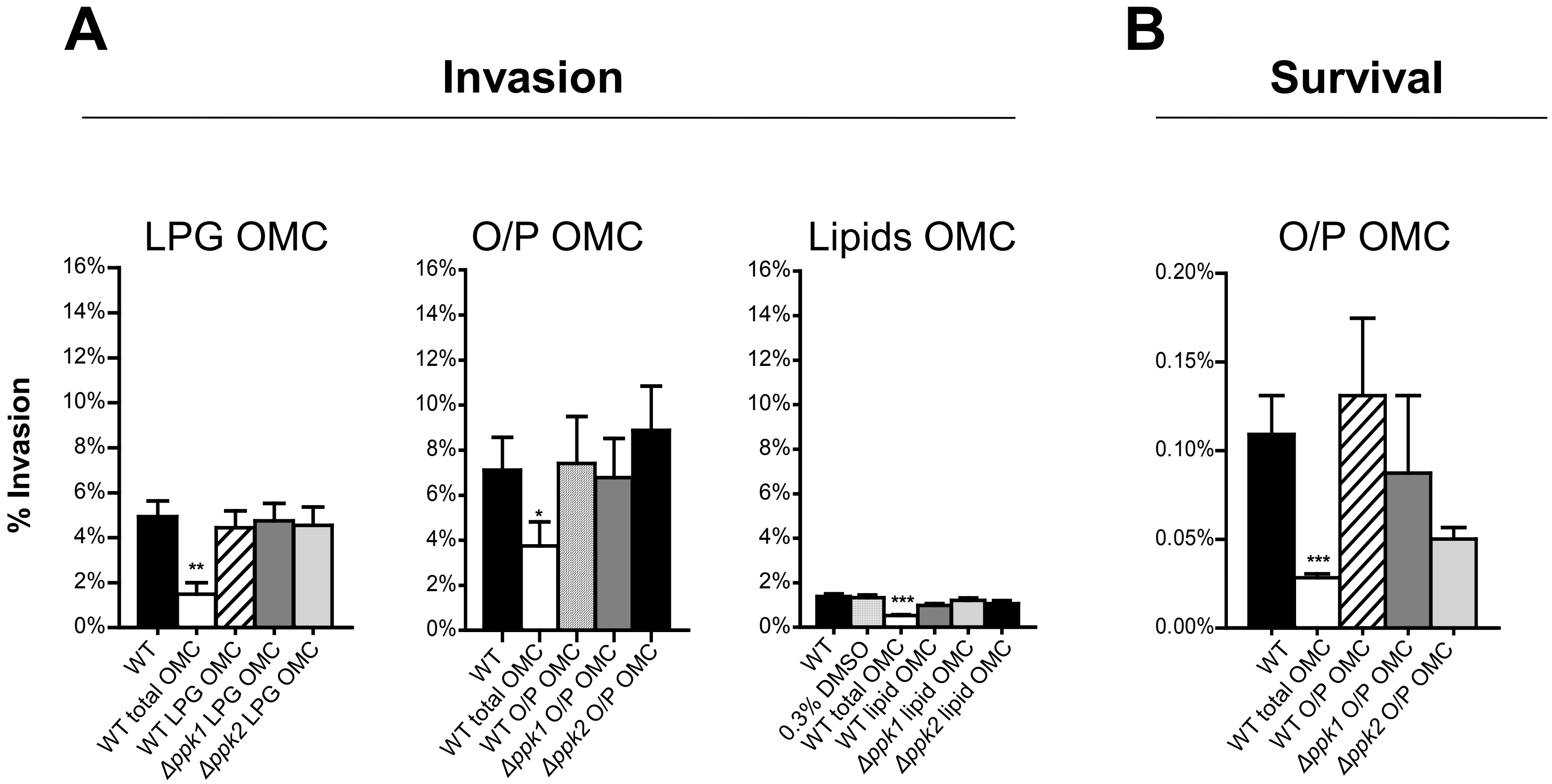

Supplemental Figure S3 (A) Effect of LPG OMC, O/P OMC, and Lipid OMC from WT, Δ*ppk1* and Δ*ppk2* on *C. jejuni* invasion of **(A)** and survival in **(B)** INT-407 cells. The total OMC was also tested with each fraction as a control using the same concentration that was used for each fraction. A 0.3% DMSO vehicle used to resuspend lipid fraction was also tested as a control. Results are presented as the mean±SEM of the number of bacteria recovered after cell lysis. Each value is the mean of at least two separate experiments performed in triplicate on different days. Asterisks (*) indicate the significant difference compared to the unexposed infected INT-407 cells (one-way ANOVA, ***P<0. 001, **P<0.01).
